# Static Analysis of Skew Functionally Graded Plate Using Novel Shear Deformation Theory

**DOI:** 10.3390/ma15134633

**Published:** 2022-07-01

**Authors:** Jitendra Singh, Ajay Kumar, Małgorzata Szafraniec, Danuta Barnat-Hunek, Barbara Sadowska-Buraczewska

**Affiliations:** 1 National Institute of Technology Patna, Patna 800005, India; jitendr1025@gmail.com (J.S.); sajaydce@gmail.com (A.K.); 2 Faculty of Civil Engineering and Architecture, Lublin University of Technology, Nadbystrzycka St. 40, 20-618 Lublin, Poland; d.barnat-hunek@pollub.pl; 3 Faculty of Civil Engineering and Environmental Sciences, Bialystok University of Technology, Wiejska St. 45A, 15-351 Bialystok, Poland; barbara.sadowska@pb.edu.pl

**Keywords:** functionally graded plates, shear deformation plate theory, static analysis of plate, finite element method

## Abstract

In this article, the static response of a functionally graded material (FGM) plate is studied via hybrid higher-order shear deformation theory which uses hyperbolic and polynomial shape functions and includes the effect of thickness stretching. The composition of the plate comprises metallic and ceramic phases. The ceramic volume fraction varies gradually along with the thickness following the power law. The mechanical properties of the FGM plate are determined by the rule of mixtures and the Mori–Tanaka homogenization scheme. The displacement fields are defined to satisfy the requirement of traction-free boundary conditions at the bottom and top surfaces of the plate surface removing the need for determination of shear correction factor. A C^0^ continuity FE model is developed for the present mathematical model. Nine-node isoparametric elements with eight nodal unknowns at each node are developed. The present model comparison with existing literature is completed and found to be coherent. Inhouse MATLAB code is developed for the present work. Sinusoidal and uniformly distributed loading is analyzed in the present work. The parametric study is undertaken to explore the effect of the side-to-thickness ratio, aspect ratio, thickness, and volume fraction index on stresses and transverse displacements.

## 1. Introduction

Functionally graded material (FGM) comes from the class of composite material with a gradual variation in their composition along a preferred direction. In 1984, FGM was introduced by Japanese material scientists as a composite material for thermal resistance [1]. The continuous grading in the FGM plates results in the continuous material property variation across its thickness from one material phase to another material phase resulting in the uniform variation in the mechanical properties between both material phases. Laminated composite structures suffer from the abrupt change in the material properties at the separation layers of the laminae, which could result in transverse shear stress discontinuity causing delamination. These abrupt changes in transverse shear stress are avoided by the FGM due to its smooth and continuous gradation across the thickness. Many engineering areas, such as marine, aircraft, and automobile sectors utilize FGM extensively. With the increasing demand for FGM, there is a need to investigate and describe their behavior efficiently with more accurate computational methods.

Various studies have been undertaken to understand the structural behavior of FGM plates. Many theories were proposed to model the static response of the FGM plates under the application of the transverse load. Three types of theories can be distinguished: three-dimensional elastic theories, discrete layer theories, and equivalent single-layer theories. Three-dimensional elastic theories offer precise displacement and stress solutions. Vel [2] proposed a precise three-dimensional solution for functionally graded plate vibrations. Kashtalyan [3] suggested three-dimensional displacement and stress solutions for a functionally graded simply supported plate subjected to transverse loads. Jin et al. [4] used three-dimensional elastic solutions for free vibrations of arbitrary thickness plates. Woodward and Kashtalyan [5] considered exact three-dimensional solutions for transversely isotropic FGM plates. For the laminated composite plate analysis, Reddy [6] suggested a simple higher-order shear deformation theory. For determining the stress and displacement of the FGM plate, Zhang et al. [7] provided semi-analytical solutions. The finite element formulation of three-dimensional elastic theories for the static analysis of the FGM plate is quite complicated, and computing the results is extremely difficult and time-consuming. Various discrete theories have been presented to predict the behavior of composite laminates and compute displacement and stresses efficiently. The local deformation theory was used by Wu and Chen [8] to analyze laminated composite plates. Cho et al. [9] employed discrete layer deformation theory to analyze the plate vibration and buckling. Icardi and Ugo [10] used an eight-node zig-zag element for the detection of stress and displacement in a laminated composite plate. The higher-order zig-zag theory was used by Ajay et al. [11] to investigate the behavior of laminated composite and sandwich shells. These theories require a different set of unknowns for each layer of the laminated composite plates. Complex kinematic boundary conditions are usually recommended in these theories, which are substantially time-consuming and complicated for implementation to get accurate results.

At present, the equivalent single layer theories are preferably employed to develop the mathematical model for computing the displacement and stresses of the FGM plate. Kirchoff and Love proposed a simple classical plate theory for displacement and stress analysis [12]. This theory does not account for the effect of shear; hence, it is used only for the analysis of thin plates. Reissner and Mindlin introduced the first-order shear deformation plate theory [13] which considered the effect of shear but required additional computation of a shear correction factor. Various higher-order shear deformation theories (HSDT) [14] have been proposed for the static analysis of FGM plates. HSDTs ensured traction-free boundary conditions on the plate’s top and bottom, eliminating the need to calculate the shear correction factor. For the static analysis of FGM plates, Talha and Singh [15] utilized polynomial functions, Touratier [16] used trigonometric functions, Thai et al. [17] recommended inverse tangent function, Soldatos [18] and Grover et al. [19] applied the hyperbolic functions, Karama et al. [20] used the exponential function, Li et al. [21] proposed the combination of trigonometric functions and polynomial functions, and Mahi et al. [22] applied the combination of hyperbolic function and polynomial functions as HSDT to describe the parabolic strain profile of the transverse shear strain.

Natarajan and Ganapathi [23] proposed HSDT with 13 unknown functions, Nelson and Lorch [24] presented HSDT with nine unknown functions, Neves et al. [25] adopted nine unknown functions, and Mantari and Soares [26] used six unknown functions for their HSDT. These theories were accurate, but computation was difficult because of the large number of unknown functions. Zenkour [27] presented a trigonometric shear deformation theory for the analysis of functionally graded plates. Using higher-order shear deformation theory, Qian et al. [28] calculated the static and dynamic deformation of a thick functionally graded elastic plate. Ferriera [29] and Gooley et al. [30] applied the MLPG method on thick FGM plates. Mechab et al. [31] proposed a two-variable improved plate theory to study the behavior of functionally graded plates by reducing the number of shear deformation functions. Mahi et al. [22] used five nodal unknowns to describe the hyperbolic shear deformation plate theory. The effect of stretching of thickness in the FGM plates and shell was investigated by Carrera et al. [32] and Habib et al. [33]. However, the equivalent single layer shear deformation theories generally use approximate mathematical formulations such as the Rayleigh–Ritz method and Fourier transformation to develop the solution, which is very specific to loading, shape, and displacement functions. Due to lack of generalization, inability to describe complicated shape and loading, and differences in findings when compared to 3-D solutions, numerical methods to simulate the behavior of functionally graded plates were developed.

To predict the behavior of the FGM plates, many researchers have chosen numerical methods. Singha et al. [34] used high precision plate bending finite elements to examine the nonlinear behavior of FGM plates. Valizadeh et al. [35] investigated the static and dynamic behavior of functionally graded plates using NURBs-based finite element analysis. Orakdogen et al. [36] studied the coupling effect of the thickness and the FGM plate bending using the finite element method. Using the finite element model, Alshorbagy et al. [37] examined the free vibration and flexure of the FGM plate. Pindera and Dunn [38] presented the effect of the thermomechanical gradient on the FGM plate using Finite element modeling. For the investigation of the FGM plate, Nguyen et al. [39] employed a triangular finite element computational algorithm. Can et al. [40] applied finite element modeling for the calculation of the buckling load. Naghdabadi and Hosseini [41] used a finite element-based model to analyze the FGM plates and shells. Talha and Singh [15] incorporated the finite element method into HSDT to describe the FGM plate’s static and free vibration responses. Taj et al. [42] modeled the FGM plate’s static response using nine node isoparametric finite elements with 10 nodal unknowns at each node.

From the literature review, it was observed that there is a need to develop the mathematical formulation with higher-order shear deformation theory considering the thickness stretching effect. Therefore, the hybrid hyperbolic and polynomial function based HSDT proposed by Mahi et al. [22] is refined by incorporating the thickness stretching effect across the thickness to develop the mathematical formulation. In the present work, after mathematical model development, its finite element model implementation is undertaken. The C^0^ finite element model is developed by the authors for simplifying the computation of the displacements and stresses. The change in strain with plate thickness is addressed using an additional polynomial function, which was not implied in the earlier theory. The convergence study is performed to compute the displacement and stress results. The model is tested by analyzing the performance with published literature. The model is used for parametric investigations such as the fluctuation of non-dimensional displacement and stresses with the volume fraction index, aspect ratio, and plate depth.

## 2. Theoretical Formulation

### 2.1. Geometrical Configuration of the FGM Plate

**Figure 1:**
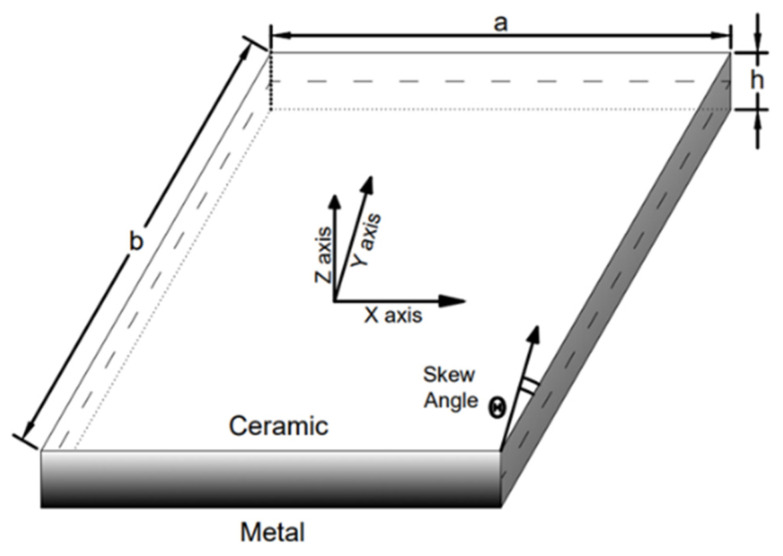
Geometry of skew functionally graded plate.

The geometrical properties of the FGM plate are described in Figure 1 with length l, width b, and thickness h. A functionally graded plate is made up of two isotropic material phases and there is a continuous gradation of the material through the thickness of the plate, with the bottom surface being the metal and the top surface being the ceramic. The coordinates x, y, and z are used in the in-plane and thickness directions, respectively. The angle of skewness is taken from the y-axis in a clockwise direction.

### 2.2. Material Homogenization Scheme

The volume fraction of the ceramic material is determined according to the power law described in Equation (1), with the index (n) and the distance from the midplane (z) of the plate.

(1) $$V_{C}\left(z\right)={\left\{\frac{1}{2}+\frac{z}{h}\right\}}^{n}$$

The value of n will range between zero and infinity where zero corresponds to the purely ceramic phase and infinity corresponds to the purely metallic phase, respectively. Vm denotes the volume fraction of the metallic phase, whereas Vc is the volume fraction of the ceramic phase, and the two are related as V_m_ = 1 − V_c_. Material properties such as modulus of elasticity, E(z), and Poisson ratio, ν(z), can be estimated as a function of distance from the midplane, z. The homogenization schemes which are used for the determination of the mechanical properties in the FGM plate are Voigt’s rule of mixtures (RM) and the Mori–Tanaka (MT) scheme. The variation in material properties such as Modulus of Elasticity and Poisson ratio across thickness is defined according to Voigt’s rule of mixture scheme given in Equations (2) and (3).

(2) $$E\left(z\right)=E_{m}+\left(E_{C}-E_{m}\right){*V}_{C}\left(z\right)$$

(3) $$\upsilon \left(z\right)=\upsilon_{m}+\left(\upsilon_{C}-\upsilon_{m}\right){*V}_{C}\left(z\right)$$

The variation in material properties such as effective bulk modulus (K) and effective shear modulus (G) across the plate’s thickness is defined according to the Mori–Tanaka scheme [43] given in Equations (4) and (5). The coefficients C_K_ and C_G_ are defined in Equation (6). The bulk modulus of isotropic material phases is calculated using Equation (7). Finally, the modulus of elasticity E_z_ and Poisson ratio υ_z_ at a particular depth are described by Equation (8).

(4) $$K_{z}=\left(K_{c}-K_{m}\right)\frac{V_{c}}{1+(1-V_{c}){*C}_{K}}+K_{m}$$

(5) $$G_{z}=\left(G_{c}-G_{m}\right)\frac{V_{c}}{1+(1-V_{c}){*C}_{G}}+G_{m}$$

(6) $$C_{K}=\frac{K_{C}-K_{m}}{K_{m}+\frac{4}{3}{*G}_{m}},{\;C}_{G}=\frac{G_{C}-G_{m}}{G_{m}+f_{1}},{\;f}_{1}=G_{m}\frac{9K_{m}+8G_{m}}{6\left(K_{m}+2G_{m}\right)}$$

(7) $$K=\frac{E}{3\left(1-2\upsilon \right)}\;G=\frac{E}{2\left(1+\upsilon \right)}$$

(8) $$E\left(z\right)=\frac{9K_{z}G_{z}}{3K_{z}+G_{z}}\;\upsilon \left(z\right)=\frac{3K_{z}-2G_{z}}{2\left(3K_{z}+G_{z}\right)}$$

### 2.3. Displacement Field Definition

The displacement fields have six nodal unknowns: *u*, *v*, *w*, *ϕ_sx_*, *ϕ_sy_*, and *ψ*, respectively. The displacements on the plate’s mid-plane along the x, y, and z-axes are denoted by *u*, *v*, and *w*, respectively. The rotation of the plate’s transverse normal about the y-axis and x-axis are *ϕ_sx_* and *ϕ_sy_* while *ψ* is the variation in the plate’s stretching deformation due to the load application. The proposed HYSD uses the shape function *f(z)* associated with *ϕ_sx_* and *ϕ_sy_* which is expressed in Equation (9) for defining the distribution of the transverse shear strain across the plate’s thickness. The shape function *g(z)* associated with *ψ* is defined in Equation (10) to account for the thickness stretching deformation of the plate.

(9) $$f\left(z\right)=\frac{h}{2}tanh\left\{\frac{2z}{h}\right\}-\frac{4z^{3}}{3h^{2}\cos h{\left(1\right)}^{2}}$$

(10) $$g\left(z\right)=\frac{3\pi }{2}\left(1-\tanh^{2}\frac{z}{h}\right)-\frac{3\pi }{2}sech^{2}\frac{1}{2}$$

The displacement fields for the in-plane *u*, *v*, and transverse w displacements are defined in Equations (11)–(13) using the shape function *f(z)* and *g(z).*

(11) $$u\left(x,y,z\right)=u_{0}\left(x,y\right)-z\frac{\partial w}{\partial x}-f\left(z\right)\phi_{sx}\left(x,y\right)$$

(12) $$v\left(x,y,z\right)=v_{0}\left(x,y\right)-z\frac{\partial w}{\partial y}-f\left(z\right)\phi_{sy}\left(x,y\right)$$

(13) $$w\left(x,y,z\right)=w_{0}\left(x,y\right)+g\left(z\right)\psi \left(x,y\right)$$

For C^0^ continuity of finite element analysis, the out-of-plane derivatives are problematic because the expression of the strain will involve terms with second-order derivatives. C^1^ continuity must be satisfied for those second-order derivative terms. The C^1^ continuity requirement is very complex and difficult to model. Hence, the out-of-plane derivatives are substituted with additional nodal unknowns to ensure that the displacement field variables are continuous within elements. These substitutions introduce artificial constraints expressed in Equation (14) in the governing equation of the plate and require application of the penalty approach during finite element formulation.

(14) $$\theta_{bx}=\frac{\partial w}{\partial x},\;\;\theta_{by}=\frac{\partial w}{\partial y}$$

The resultant modified displacement fields for in-plane displacements are expressed in Equations (15) and (16).

(15) $$u\left(x,y,z\right)=u_{0}\left(x,y\right)-z\theta_{bx}-f\left(z\right)\phi_{sx}\left(x,y\right)\;\;$$

(16) $$v\left(x,y,z\right)=v_{0}\left(x,y\right)-z\theta_{by}-f\left(z\right)\phi_{sy}\left(x,y\right)$$

### 2.4. Strain–Displacement Relationship

The strain–displacement relationships which are obtained from the differentiation of the displacement field equations are described as follows:

(17) $$\in_{xx}=\frac{\partial u_{o}}{\partial x}-z\frac{\partial \theta_{bx}}{\partial x}-f\left(z\right)\frac{\partial \phi_{sx}}{\partial x}\;\;\in_{yy}=\frac{\partial v_{o}}{\partial y}-z\frac{\partial \theta_{by}}{\partial y}-f\left(z\right)\frac{\partial \phi_{sy}}{\partial y}\;\;\in_{zz}=g^{\prime }\left(z\right)\psi \;\;\;\;\;\;\;\gamma_{xy}=\frac{\partial u_{o}}{\partial y}+\frac{\partial v_{o}}{\partial x}-z\left\{\frac{\partial \theta_{bx}}{\partial y}+\frac{\partial \theta_{by}}{\partial x}\right\}-f\left(z\right)\frac{\partial \phi_{sx}}{\partial y}\frac{\partial \phi_{sy}}{\partial x}\}\;\;\;\gamma_{xz}=-\theta_{bx}+\left\{\frac{\partial f_{s}}{\partial z}\theta_{sx}+\frac{\partial w_{o}}{\partial x}\right\}+g\left(z\right)\frac{\partial \psi }{\partial x}\;\gamma_{yz}=-\theta_{by}+\left\{\frac{\partial f_{s}}{\partial z}\theta_{sy}+\frac{\partial w_{o}}{\partial y}\right\}+g\left(z\right)\frac{\partial \psi }{\partial x}$$

### 2.5. Constitutive Relationship

The linear constitutive relationship between the stresses and the strain is given by the constitutive matrix [44] expressed in Equation (19). The effective material properties such as *E(z)* and *ν(z)* are defined according to Voigt’s rule of mixtures or the Mori–Tanaka scheme [43]. *Q_ij_* described in Equation (18) varies with the fluctuation in *E(z)* and *ν(z)* across the plate thickness.

(18) $$Q_{11}=Q_{22}=Q_{33}=\frac{E\left(z\right)\left(1-v{\left(z\right)}^{2}\right)}{1-3v{\left(z\right)}^{2}+2v{\left(z\right)}^{3}}\;\;Q_{12}=Q_{13}=Q_{23}=\frac{E\left(z\right)v\left(z\right)\left(1+v\left(z\right)\right)}{1-3v{\left(z\right)}^{2}+2v{\left(z\right)}^{3}}\;Q_{44}=Q_{55}=Q_{66}=\frac{E\left(z\right)}{2\left(1+v\left(z\right)\right)}\;$$

(19) $$\left[\begin{array}{c} \sigma_{xx}\\ \sigma_{yy}\\ \sigma_{zz}\\ \tau_{xy}\\ \tau_{xz}\\ \tau_{yz} \end{array}\right]=\left[\begin{array}{cccccc} Q_{11} & Q_{12} & Q_{13} & 0 & 0 & 0\\ Q_{12} & Q_{22} & Q_{23} & 0 & 0 & 0\\ Q_{13} & Q_{23} & Q_{33} & 0 & 0 & 0\\ 0 & 0 & 0 & Q_{44} & 0 & 0\\ 0 & 0 & 0 & 0 & Q_{55} & 0\\ 0 & 0 & 0 & 0 & 0 & Q_{66} \end{array}\right]\left[\begin{array}{c} \epsilon_{xx}\\ \epsilon_{yy}\\ \epsilon_{zz}\\ \gamma_{xy}\\ \gamma_{xz}\\ \gamma_{yz} \end{array}\right]$$

## 3. Finite Element Formulation

The governing differential equation of the plate can be obtained using the application of the principal of virtual work on the strain energy U, external work W, and artificial constraint C of the FGM plate system as described in Equation (20).

(20) $$\oint^{\;}\delta \left(U+C-W_{ext}\right)\partial \Omega =0$$

The strain energy of the system can be expressed by Equation (21). The strain vectors can be represented as the product of thickness coordinate matrix [*H*], differential operator matrix [B] described in Appendix A, and mid-plane displacements {*X_o_*} described in Equation (22). Similarly, the work done by the external load is defined in Equation (23). The external load vector *q* = [0 0 q_o_ 0 0 0 0 0] in which *q_o_* (x, y) corresponds to the transverse load acting on the midplane.

(21) $$U=\oint^{\;}\sigma_{ij}{}^{T}\epsilon_{ij}\partial \Omega$$

(22) $$\epsilon =\left[H\right]\left[B\right]\left\{X_{o}\right\}$$

(23) $$W_{ext}=\oint^{\;}q^{T}X\;\partial A$$

The nine-node isoparametric Lagrangian shape functions were used in the development of the isoparametric finite element for the analysis of the FGM plate. The use of a nine-node element allows (3 × 3) a full integration scheme to be implemented and this results in improvement in the accuracy of the results. The midplane displacement can be interpolated using the isoparametric shape functions described in Equation (24).

(24) $$\left\{X_{o}\right\}=\overset{9}{\underset{i=1}{\sum }}N_{i}X_{oi}$$

The material rigidity matrix [D] is calculated using the constitutive matrix given in Equation (19) and thickness coordinate matrix [H]. This matrix helps in the implementation of the proposed HSDT as an equivalent single layer theory to convert the 3D domain to the 2D domain for the analysis.

(25) $$\left[D\right]=\int_{z=-h/2}^{z=h/2}{\left[H\right]}^{T}\left[Q_{ij}\right]\left[H\right]\partial z$$

Finally, the application of Equations (21)–(25) in Equation (20) results in the governing equation for the static analysis of the FGM plate described in Equation (26). The stiffness matrix [K], constraint stiffness matrix [K_c_], and force matrix [F] can be derived from Equation (26) to establish the relationship between load and displacement expressed in Equation (28). Since the penalty approach was used for the finite element formulation, the effect of the artificial constraints on the displacements is negligible and the relationship K X_o_ = F holds true for the present static analysis.

(26) $$\oint^{\;}\delta {\left\{X_{o}\right\}}^{T}\left[B^{T}\right]\left[D\right]\left[B\right]\left\{X_{o}\right\}\partial A+C-\oint^{\;}\delta {\left\{X_{o}\right\}}^{T}{\left\{N\right\}}^{T}\left\{N\right\}\left\{q\right\}\partial A=0$$

(27) $$\left[K\right]=\oint^{\;}\left[B^{T}\right]\left[D\right]\left[B\right]\partial A,\;\;\left[F\right]=\oint^{\;}{\left\{N\right\}}^{T}\left\{N\right\}\left\{q\right\}\partial A$$

(28) $$\left\{\left[K\right]+\gamma_{c}\left[K_{c}\right]\right\}\left\{X_{o}\right\}=\left[F\right]$$

## 4. Results

### 4.1. Model Convergence and Validation

In this section, the static analysis is performed for the calculation of displacement and stresses of the FGM plate for different types of loading, plate geometries, and boundary conditions. The mechanical properties of the material used in FGM plate are listed in Table 1.

The different boundary conditions of the plate are described in Table 2. Here, all side simply supported (SSSS), all sides clamped (CCCC), two sides simply supported two side clamped (SCSC), and two sides simply supported two sides free (SFSF) are defined by restraining the various displacements and rotations as the edges.

The FE model used for the static analysis is developed from the governing equations derived from principle of virtual work based on the proposed novel HSDT. The nine-noded Lagrangian isoparametric shape functions are utilized to write the in-house code for the FE formulation using MATLAB programming language. The FE analysis is performed for a homogeneous isotropic square simply supported plate with a/h = 10 for mesh convergence and assessment of FE model performance. The modulus of elasticity, E = 210 GPa, and Poisson ratio, ν = 0.3. The plate is subjected to a uniformly distributed load. The different mesh sizes are used for the convergence studies and findings in terms of non-dimensional central deflections and stresses defined by Equations (29)–(31) are expressed in Table 3. The model is compared with the results of Shimpi et al. [14] and Hebali et al. [33] for validation and accuracy. It was observed that sufficient convergence can be achieved for a mesh size with 11X11 elements for displacement while convergence in the stresses requires finer mesh sizes because stresses are derived quantities.

(29) $$w^{1}=100E*\frac{h^{3}}{a^{4}}\;*\frac{w\left(\frac{a}{2},\frac{b}{2},0\right)}{q_{0}}\;,\;\;\sigma_{xx}{}^{1}=\frac{h^{2}}{a^{2}}*\frac{\sigma_{xx}\;\left(\frac{a}{2},\frac{b}{2},\frac{h}{2}\right)}{q_{0}}\;\;\;\;\sigma_{yy}{}^{1}=\frac{h^{2}}{a^{2}}*\frac{\sigma_{yy}\left(\frac{a}{2},\frac{b}{2},\frac{h}{2}\right)}{q_{0}},\;\;\;\tau_{xy}{}^{1}=\frac{h^{2}}{a^{2}}*\frac{\tau_{xy}\left(0,0,\frac{h}{2}\right)}{q_{0}}\;\tau_{xz}{}^{1}=\frac{h}{a}*\frac{\tau_{xz}\left(0,\frac{b}{2},0\right)}{q_{0}},\;\;\;\;\sigma_{yz}{}^{1}=\frac{h}{a}*\frac{\tau_{xz}\left(\frac{a}{2},0,0\right)}{q_{0}}$$

The volume fraction of the ceramic phase decreases with index (*n*) and it has a significant effect on the performance of the FGM plate. The sinusoidal loading is applied on a thin (a/h = 10) simply supported square FGM plate. The mechanical properties of the Al/Al2O3 FGM is estimated using RM and MT schemes. The volume fraction index is varied from ceramic to metallic phase to examine its effect on the displacement and stresses of the plate. The results are compared with the results of Zenkour [27] where the thickness stretching effect is ignored and Hebali et al. [33], where the effect of thickness stretching is considered. The RM method captures axial stress results accurately while giving the lower plate’s central deflection values. The MT scheme captured transverse shear stresses effectively but yielded higher values of deflection. Table 4 presents the results for comparison for non-dimensional displacement, axial stress, and transverse shear stress defined in Equation (30). The gradual increase in displacement with volume fraction index can be observed. The decrease in ceramic volume fraction with increase in volume fraction index decreases the flexural rigidity of the plate.

(30) $$w^{2}=10E_{c}*\frac{h^{3}}{a^{4}}*\frac{w\left(\frac{a}{2},\frac{b}{2},0\right)}{q_{0}}\;\sigma_{xx}{}^{2}=\frac{h}{a}*\frac{\sigma_{xx}\left(\frac{a}{2},\frac{b}{2},\frac{h}{2}\right)}{q_{0}}\;,\tau_{xz}{}^{2}=\frac{h}{a}*\frac{\tau_{xz}\left(0,\frac{b}{2},0\right)}{q_{0}}\;$$

The effect of material homogenization methods such as the Mori–Tanaka scheme and Voigt’s rule of mixture is investigated using results derived by Ferreira et al. [29] and Qian et al. [28]. A square plate made of Al/ZrO2-1 FGM is analyzed for uniformly distributed load and simply supported boundary condition. The side-to-thickness ratio (a/h) and volume fraction index (n) are varied. The FE analysis is performed, and results are shown in Table 5 for comparison with the non-dimensional central deflection of the FGM plate expressed in Equation (31). The results of non-dimensional central deflection show excellent agreement with reference results for both the RM and MT schemes with RM producing slightly lower values and the MT scheme yielding higher values.

(31) $$w^{3}=E_{m}*\frac{h^{3}}{a^{4}}\;*\frac{w\left(\frac{a}{2},\frac{b}{2},0\right)}{q_{0}}\;\;\;\;$$

The effect of variation in the side-to-thickness ratio is examined to elaborate the thickness stretching effect which was incorporated in development of the FE model of the square Al-ZrO2-1 FGM plate. The effective mechanical properties across the thickness are defined by the Mori–Tanaka scheme. The uniformly distributed load is applied on a simply supported square FGM plate with varying side-to-thickness ratios and power law indices. The results are presented in Table 6 for comparison with the results given by Ferreira et al. [29] and Qian et al. [28] for the non-dimensional central deflection of the FGM plate given in Equation (31).

Similarly, another case of the square simply supported Al/Al_2_O_3_ FGM plate is taken. The Voigt’s RM method is used to define effective mechanical properties of the plate. The FGM plate is subjected to sinusoidal loading for thick, moderately thick, and thin plates. Table 7 shows the non-dimensional central deflection and axial stress, which can be compared with the findings of Carrera et al. [32], Neves et al. [45], and Hebali et al. [33] for sinusoidal load and non-dimensional central deflection and axial stress defined in Equation (32). The results predicted by the present theory strongly match the results of the existing literature for thin plates.

(32) $$w^{4}=10E_{c}*\frac{h^{3}}{a^{4}}*\frac{w\left(\frac{a}{2},\frac{b}{2},0\right)}{q_{0}}\;\;\;\sigma_{xx}{}^{4}=\frac{h}{a}*\frac{\sigma_{xx}\left(\frac{a}{2},\frac{b}{2},\frac{h}{3}\right)}{q_{0}}\;\;\;$$

The deflection is restrained by the clamped boundary conditions while the free edges tend to increase the deflection. The effect of various boundary conditions on the displacement and stresses defined in Equation (33) is studied. The mechanical properties of Al/ZrO_2_-2 FGM is defined by the MT scheme. A thick (a/h = 5) square FGM plate subjected to a uniformly distributed load is used for analysis. The analysis results are compared with multiquadric and thin plate splines results of Gilhooley et al. [30]. The results presented in Table 8 demonstrate the expected lower values of displacement for clamped boundary conditions and higher values for FGM plates with free edges.

(33) $$w^{5}=\frac{100E_{m}}{12\left(1-\nu^{2}\right)}*\frac{h^{3}}{a^{4}}*\frac{w\left(\frac{a}{2},\frac{b}{2},0\right)}{q_{0}},\sigma_{xx}{}^{5}=\frac{h^{2}}{a^{2}}*\frac{\sigma_{xx}\left(\frac{a}{2},\frac{b}{2},\pm \frac{h}{2}\right)}{q_{0}}\;\;\;$$

The stiffening effect of the skew simply supported FGM plate is explored for various skew angles. The mechanical properties of the Ti_6_Al_4_V/Si_3_N_4_ FGM plate are described by the MT scheme. The static analysis is performed for thick, moderately thick, and thin skew FGM plates all having edges of equal length. Uniformly distributed load is applied and the plate’s non-dimensional central deflection defined by Equation (34) is estimated for various skew angles and side-to-thickness ratios. The deflection of the plate is compared with Liew and Han [46] for isotropic ceramic phase. It is observed in Table 9 that the increase in the skew angle has an overall stiffening effect on the values of the displacement and larger skew angles give better performance. The effect of decreasing the stiffer ceramic volume fraction with the increase in volume fraction index yields higher displacement values.

(34) $$w^{6}=\frac{1600E_{m}}{12\left(1-\nu^{2}\right)}*\frac{h^{3}}{a^{4}}*\frac{w\left(\frac{a}{2},\frac{b}{2,0}\right)}{q_{0}}\;$$

The clamped edges of the skew FGM plate remove all the complexities arising from ambiguities about rotational displacement at the inclined edges. The effective mechanical properties of the Ti_6_Al_4_V/Si_3_N_4_ FGM plate are described by the RM method. All sides of the FGM plate are of equal length 2a and 2a/h = 100. The non-dimensional central deflection of the FGM plate is estimated for various skew angles. The results of entirely ceramic isotropic plate are compared with Butalia et al. [47] in Table 10 for non-dimensional central displacement defined by Equation (34). The clamped edges produce smaller displacement because angular displacements are restrained at the edges.

### 4.2. Parametric Studies

The effect of changing the boundary conditions, volume fraction index, side-to-thickness ratio, and aspect ratio on the FGM plate is studied. The finite element model for the FGM plate under the application of a uniformly distributed load is studied. The metallic phase of the FGM plate is made up of aluminum while the ceramic phase is made with Alumina. The plate’s non-dimensional central deflection, axial stress, and transverse shear stresses are defined in Equation (35).

(35) $$w=10E_{c}*\frac{h^{3}}{a^{4}}*\frac{w\left(\frac{a}{2},\frac{b}{2},0\right)}{q_{0}}\;\;\sigma_{xx}=\frac{h}{a}*\frac{\sigma_{xx}\left(\frac{a}{2},\frac{b}{2},\frac{h}{2}\right)}{q_{0}}\;,\;\;\;\tau_{xz}=\frac{h}{a}*\frac{\tau_{xz}\left(0,\frac{b}{2,0}\right)}{q_{0}}$$

**Figure 2:**
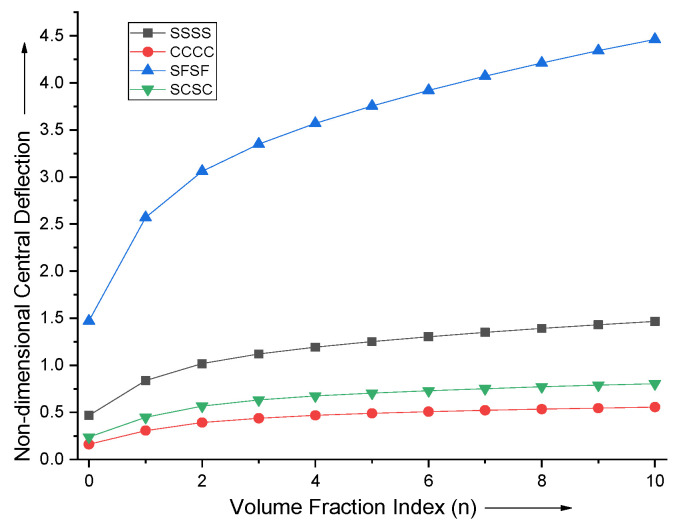
Effect of vole fraction index (n) on non−dimensional central deflection of the plate.

The effect of volume fraction index n on the non-dimensional central deflection of the FGM plate for various boundary conditions is shown in Figure 2. It can be seen that the deflection increases with the decrease in stiffer ceramic material phase volume. The deflection is maximum for the simply supported free boundary condition and it is minimum for the all sides clamped condition.

**Figure 3:**
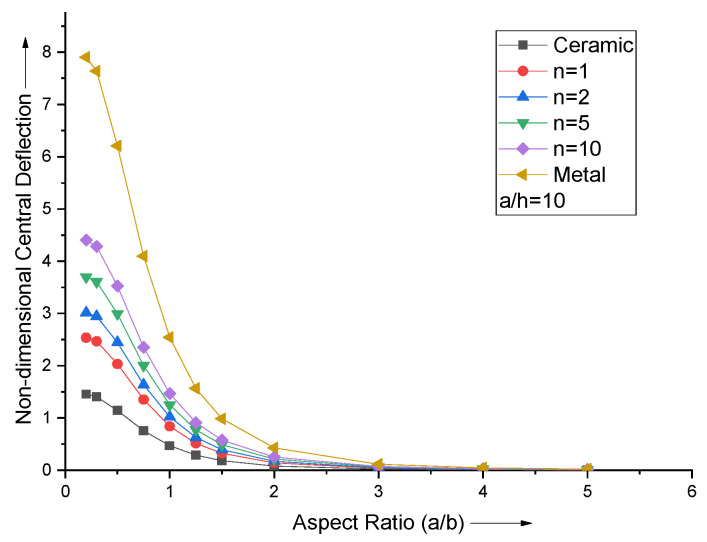
Effect of aspect ratio (a/b) on non−dimensional central deflection of the plate.

The effect of the aspect ratio of the FGM plate is investigated by varying aspect ratio and volume fraction index fixing a/h = 10. The dependence of plate’s non-dimensional central deflection on the a/b ratio is depicted in Figure 3. It is observed that the effect of change in aspect ratio a/b becomes negligible after a/b = 3. It is more prominent when a/b < 1.

**Figure 4:**
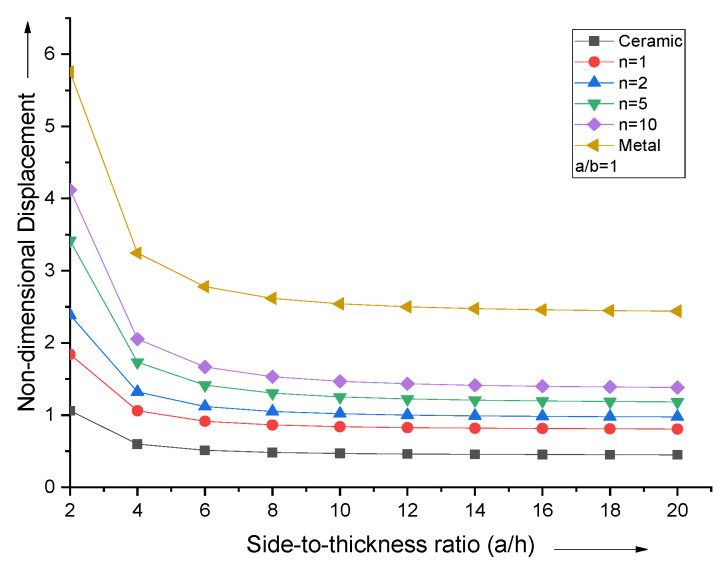
Effect of side−to−thickness ratio (a/h) on non−dimensional central deflection of the plate.

For various values of the volume fraction index, the effect of changing the side-to-thickness ratio (a/h) on the non-dimensional central deflection for a square simply supported FGM plate is shown in Figure 4. After a/h = 10, it is observed that the non-dimensional centroidal displacement curves follow a parallel line pattern.

**Figure 5:**
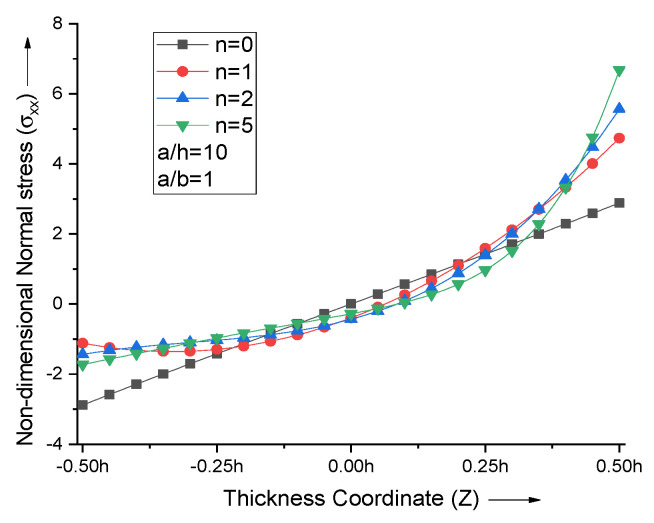
Effect of volume fraction index (n) on non−dimensional normal stresses of the plate.

When the volume fraction index is varied, the non-dimensional normal stress curves attain the extremum near the top and bottom surfaces of the FGM plate, as shown in Figure 5. The neutral axis plane shifts towards the top metallic portion for lower values of n and then comes back to midplane for higher n values.

**Figure 6:**
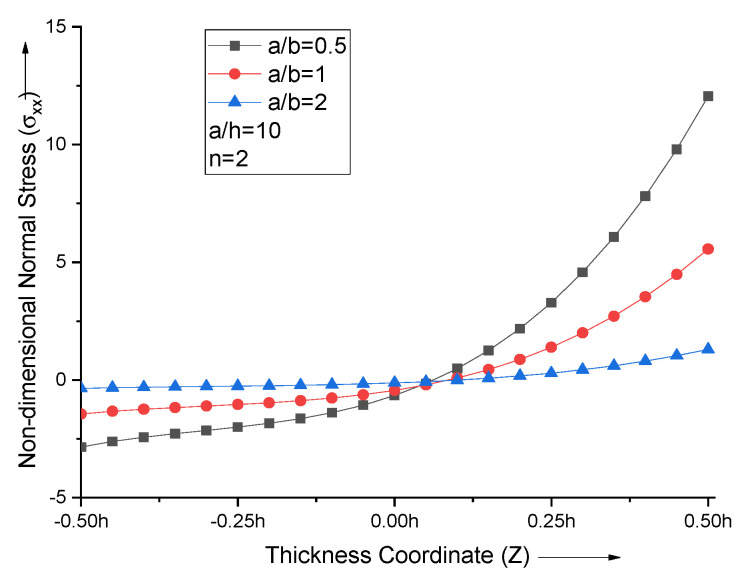
Effect of aspect ratio (a/b) on non−dimensional normal stresses of the plate.

When the aspect ratio is varied, the extremum of non-dimensional normal stress at the top and bottom surfaces of the FGM plate decreases with the increase in aspect ratio. The non-dimensional normal stress curves attain zero at Z = 0.075 for all aspect ratios of the FGM plate with *n* = 2, as shown in Figure 6.

**Figure 7:**
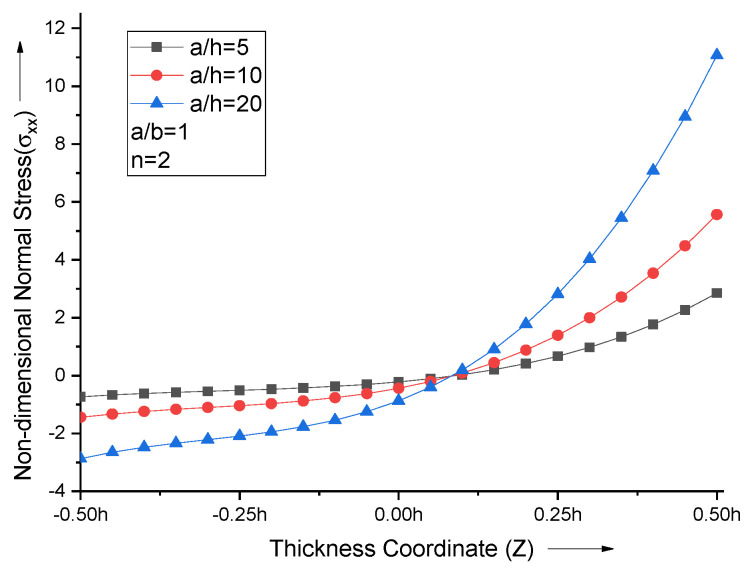
Effect of side−to−thickness ratio (a/h) on non−dimensional normal stresses of the plate.

The increase in the side-to-thickness ratio (a/h) leads to an increase in the maximum normal stress value. For all the values of the side-to-thickness ratio with *n* = 2, non-dimensional normal stress curves attain zero at Z = 0.075, as shown in Figure 7.

**Figure 8:**
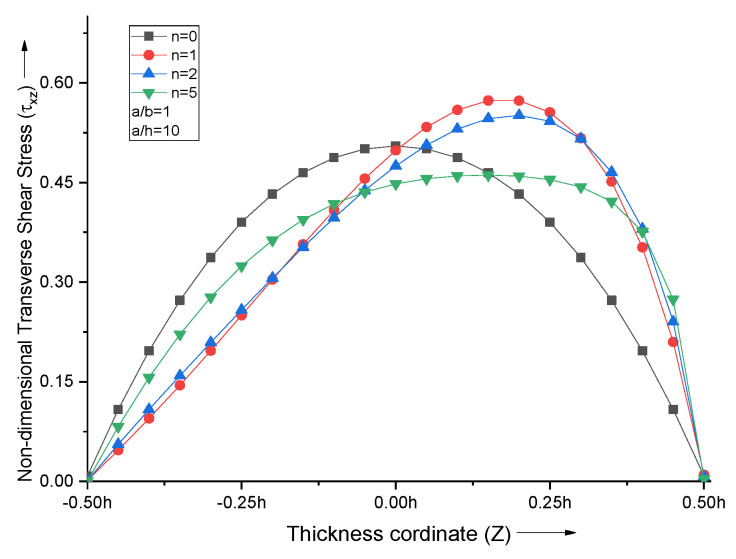
Effect of volume fraction index (n) on non−dimensional transverse shear stresses of the plate.

The non-dimensional transverse shear stress curves follow the parabolic profile, which attains a maximum value near the middle portion of the FGM plate, as shown in Figure 8. The point of maxima shifts towards the top metallic portion for lower values of n and then comes back to the midplane for higher n values.

**Figure 9:**
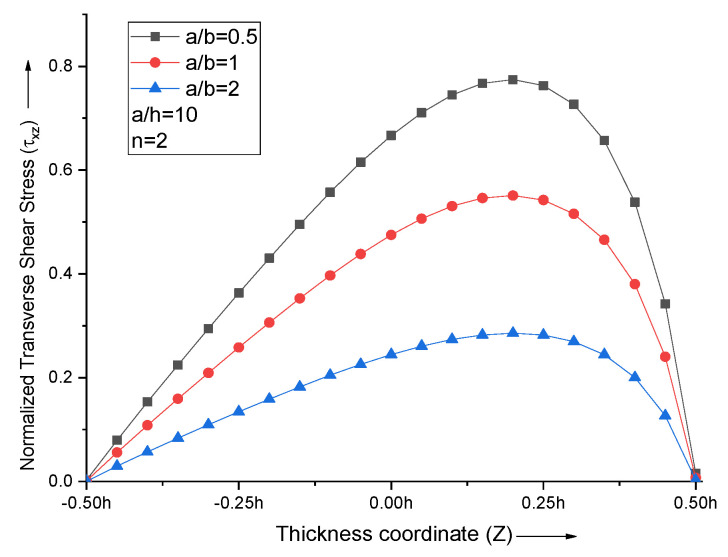
Effect of aspect ratio (a/b) on non−dimensional transverse shear stresses of the plate.

An increase in the aspect ratio (a/b) decreases the maximum transverse shear stress. For all the aspect ratios with *n* = 2, non-dimensional transverse shear stress curves acquire maxima at Z = 0.2, as shown in Figure 9.

**Figure 10:**
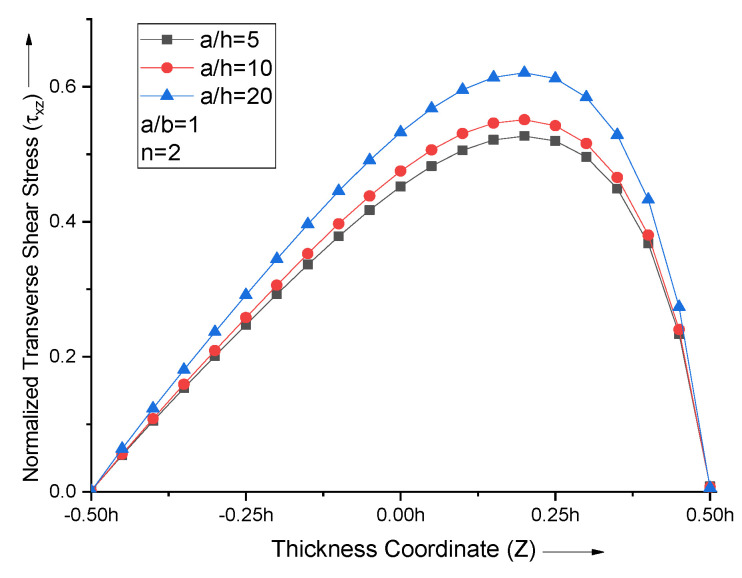
Effect of side−to−thickness ratio (a/h) on non−dimensional normal stresses of the plate.

With the increase in the side-to-thickness ratio (a/h), the maximum transverse shear stress value increases. The transverse shear stress curves for all the values of the side-to-thickness ratio with *n* = 2 attain maxima Z = 0.2, as shown in Figure 10.

**Figure 11:**
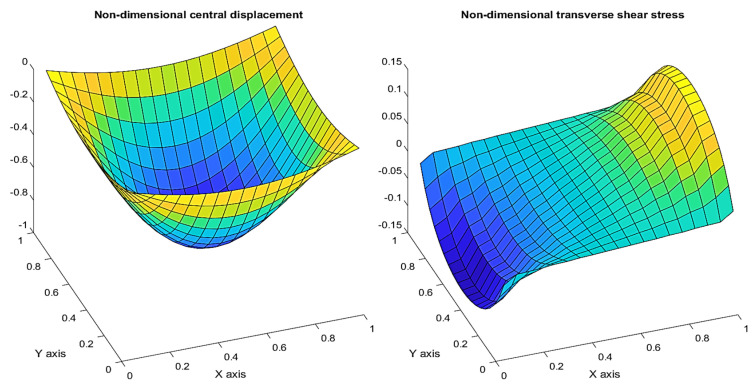
Variation in the non−dimensional displacement (w) and transverse shear stress (τ_xz_) across the FGM plate.

The variation in the displacement across the simply supported square FGM plate for *n* = 1 can be shown in Figure 11. It can be seen that the plate’s deflection obeys the boundary condition and the maximum displacement can be observed at the center of the plate. The transverse shear stress appears to give a maximum response at the mid-point of the edge which allows the corresponding shear rotation at the edge.

## 5. Conclusions

The nine-node isoparametric element-based FE model was formulated on refined hybrid polynomial and hyperbolic shape function-based higher-order shear deformation theory to analyze the FGM plate for various loading conditions. The thickness stretching effect was incorporated. The principle of virtual work was applied for the evolution of elementary stiffness and force matrix. The finite element analysis results were compared with existing literature, and the performance of the finite element model was found to be satisfactory. The model was utilized for parametric studies. The variation in boundary conditions, aspect ratio, side-to-thickness ratio, skewness, and volume fraction index was completed. The following observations were made regarding non-dimensional central deflection and stresses.

- Non-dimensional central deflection of the plate decreases with increase in volume fraction index (*n*) due to decrease in stiffer ceramic fraction.
- When both the Mori–Tanaka scheme and Voigt’s rule of mixture method are employed to define the effective material properties of the FGM plate, the Mori–Tanaka scheme gives more deflection.
- The plate’s non-dimensional central deflection is maximum for the SFSF boundary condition and minimum for the CCCC boundary condition.
- Variation in the plate’s non-dimensional central deflection with aspect ratio is negligible when a/b > 3.
- With the variation in side-to-thickness ratio, the plate’s non-dimensional central deflection shows a parallel line pattern when a/h > 10.
- The non-dimensional normal stress curves attain extremum near the top and bottom surfaces of the FGM plate. When the volume fraction index increases, the maximum value of axial stress also increases.
- When the volume fraction index is greater than zero, the peak of the parabolic profile of non-dimensional transverse shear stress shows a shift towards the top surface of the plate.
- Non-dimensional normal stress decreases with an increase in the aspect ratio, and its value increases with the increase in the side-to-thickness ratio. It has the same location for the zero stress value, irrespective of a/h or a/b ratio for a particular volume fraction index.
- The non-dimensional transverse shear stress decreases with an increase in the aspect ratio, and it increases with the increment in the side-to-thickness ratio. It attains the maxima at the same location irrespective of a/h or a/b ratio for a particular volume fraction index.
- The non-dimensional central deflection of the skewed FGM plate decreases with increase in skew angles because of the stiffening effect of larger skew angles.

## Figures and Tables

**Table 1:** Mechanical properties of the material used in functionally graded material.

Material	Chemical Formula	E (GPa)	ν
Aluminum	Al	70	0.3
Alumina-1	Al_2_O_3_	380	0.3
Zirconia-1	ZrO_2_-1	151	0.3
Zirconia-2	ZrO_2_-2	200	0.3
Titanium alloy	Ti_6_Al_4_V- Si_3_N_4_	105.7	0.3
Silicon nitride	Si_3_N_4_	322.27	0.24

**Table 2:** Definition of the various boundary condition for the analysis of the plate.

Boundary	Edge	Displacement				Rotation			
Condition	Definition	u	v	w	ψ	θ_x_	ϕ_x_	θ_y_	ϕ_y_
SSSS	X = 0,a	--	0	0	0	0	0	--	--
	Y = 0,b	0	--	0	0	--	--	0	0
CCCC	X = 0,a	0	0	0	0	0	0	0	0
	Y = 0,b	0	0	0	0	0	0	0	0
SCSC	X = 0,a	--	0	0	0	0	0	--	--
	Y = 0,b	0	0	0	0	0	0	0	0
SFSF	X = 0,a	--	0	0	0	0	0	--	--
	Y = 0,b	--	--	--	--	--	--	--	--

**Table 3:** Non-dimensional central deflection and stresses in a homogeneous isotropic square plate subjected to the uniformly distributed load.

Theory	w^1^	σ_xx_^1^	σ_yy_^1^	τ_xy_^1^	τ_xz_^1^	τ_yz_^1^
Present (4 × 4)	4.6144	0.30032	0.30032	0.1915	0.57325	0.57325
Present (6 × 6)	4.6308	0.30166	0.30166	0.19363	0.54587	0.54587
Present (9 × 9)	4.6342	0.30167	0.30167	0.19445	0.52517	0.52517
Present (11 × 11)	4.6348	0.30176	0.30176	0.19462	0.51848	0.51848
Present (13 × 13)	4.6350	0.30183	0.30183	0.19471	0.51443	0.51443
Shimpi et al. [14]	4.625	0.307	0.307	0.195	0.505	0.505
Hebali et al. [33]	4.631	0.276	0.276	0.197	0.481	0.481

**Table 4:** Non-dimensional central deflection and axial stress of an FGM plate subjected to uniformly distributed load under SSSS.

	w^2^				σ_xx_^2^			τ_xz_^2^		
	Ref ^1^	Ref ^2^	(RM)	(MT)	Ref ^1^	Ref ^2^	(RM)	Ref ^1^	Ref ^2^	Present (MT)
*n*	ϵ_zz_ ≠ 0	ϵ_zz_ = 0	ϵ_zz_ ≠ 0	ϵ_zz_ ≠ 0	ϵ_zz_ ≠ 0	ϵ_zz_ = 0	ϵ_zz_ ≠ 0	ϵ_zz_ ≠ 0	ϵ_zz_ = 0	ϵ_zz_ ≠ 0
Ceramic	0.2937	0.296	0.29391	0.29391	1.9076	1.9955	2.086	0.2327	0.2462	0.25602
1	0.5689	0.5889	0.53005	0.654	2.9105	3.087	3.3937	0.2326	0.2462	0.25208
2	0.722	0.7573	0.64552	0.77639	3.4198	3.6094	3.9778	0.2122	0.2265	0.23829
3	0.7972	0.8377	0.71086	0.85081	3.6729	3.8742	4.2988	0.1958	0.2107	0.23087
4	0.8413	0.8819	0.75701	0.90636	3.8569	4.0693	4.542	0.1877	0.2029	0.22828
5	0.8729	0.9118	0.79436	0.95163	4.0273	4.2488	4.758	0.1861	0.2017	0.22824
6	0.8983	0.9356	0.82664	0.99015	4.1954	4.4244	4.9602	0.1884	0.2041	0.22932
7	0.9211	0.9562	0.85549	1.0237	4.3619	4.5971	5.1522	0.1923	0.2081	0.23075
8	0.9416	0.975	0.88176	1.0535	4.5251	4.7661	5.3351	0.1968	0.2124	0.2322
9	0.9606	0.9925	0.90596	1.0801	4.6846	4.9303	5.5094	0.2009	0.2164	0.23352
10	0.9793	1.0089	0.92842	1.1041	4.8388	5.089	5.6755	0.2046	0.2198	0.23468
Metal	1.5942	1.607	1.5955	1.5955	1.9076	1.9955	2.086	0.2327	0.2462	0.25602

^1^ Zenkour [27], ^2^ Hebali et al. [33].

**Table 5:** Non-dimensional central deflection (w^3^) of the simply supported square FGM plate effective elastic properties computed by Voigt’s rule of mixtures and Mori–Tanaka scheme.

		Voigt’s Rule of Mixtures Scheme			Mori–Tanaka Scheme		
a/h	*n*	Ref ^1^	Ref ^2^	Present	Ref ^1^	Ref ^2^	Present
5	0	0.02477	0.02436	0.024299	0.02477	0.02436	0.024299
	0.5	0.03135	--	0.030377	0.03293	--	0.031789
	1	0.03515	0.03634	0.03367	0.03666	0.03634	0.035082
	2	0.03883	0.03976	0.037008	0.04009	0.03976	0.03836
	Metal	0.05343	0.05253	0.052416	0.05343	0.05253	0.052416
20	0	0.0208	0.02118	0.020768	0.0208	0.02118	0.020768
	0.5	0.0265	--	0.026144	0.0279	--	0.027298
	1	0.0297	0.0315	0.028847	0.0309	0.0315	0.029906
	2	0.0324	0.03395	0.031286	0.0330	0.03395	0.03228
	Metal	0.0448	0.0458	0.0448	0.0448	0.0458	0.0448

^1^ Ferreira et al. [29], ^2^ Qian et al. [28].

**Table 6:** Effect of the variation in a/h ratio on the non-dimensional central displacement (w).

Theory	*n*	a/h = 5	a/h = 15	a/h = 25	a/h = 45	a/h = 75	a/h = 125
Ref ^1^	0	0.02436	0.02115	0.02123	0.02158	0.0219	0.02225
	1	0.03634	0.03152	0.03158	0.03203	0.03252	0.03304
	2	0.03976	0.03401	0.03404	0.03456	0.03501	0.03562
	Metal	0.05252	0.04583	0.04569	0.04655	0.04728	0.04802
Ref ^2^	0	0.02476	0.0209	0.02062	0.02057	0.02062	0.02069
	1	0.03666	0.03103	0.03061	0.03054	0.03061	0.03072
	2	0.04009	0.03354	0.03305	0.03295	0.03302	0.03314
	Metal	0.05342	0.0451	0.04448	0.04437	0.04448	0.04464
Present	0	0.024299	0.020957	0.020678	0.020554	0.020506	0.020484
	1	0.035082	0.030183	0.029773	0.029589	0.029518	0.029484
	2	0.03836	0.032604	0.032125	0.031913	0.031831	0.031793
	Metal	0.052416	0.045207	0.044606	0.044338	0.044235	0.044186

^1^ Ferreira et al. [29], ^2^ Qian et al. [28].

**Table 7:** The variation in non-dimensional central displacement and axial stress subjected to sinusoidal loading.

		w^4^			σ^4^_xx_		
*n*	Theory	a/h = 4	a/h = 10	a/h = 100	a/h = 4	a/h = 10	a/h = 100
1	Ref ^1^	0.7289	0.589	0.5625	0.7856	2.0068	20.149
	Ref ^2^	0.6997	0.5845	0.5624	0.5925	1.4945	14.969
	Ref ^3^	0.691	0.5686	0.50526	0.5952	1.4954	16.8589
	Present	0.65189	0.53005	0.50418	0.67017	1.6649	16.4934
4	Ref ^1^	1.1673	0.8828	0.8286	0.5986	1.5874	16.047
	Ref ^2^	1.1178	0.875	0.8286	0.4404	1.1783	11.932
	Ref ^3^	1.0964	0.8413	0.7052	0.4507	1.1779	14.3359
	Present	1.0151	0.75701	0.7037	0.53761	1.3937	13.9392
10	Ref ^1^	1.3925	1.009	0.9361	0.4345	1.1807	11.989
	Ref ^2^	1.349	0.875	0.8286	0.3227	1.1783	11.932
	Ref ^3^	1.3333	0.9791	0.9114	0.3325	0.8889	8.9977
	Present	1.2799	0.92842	0.85713	0.37709	0.99298	9.952

^1^ Carrera et al. [32], ^2^ Neves et al. [45], ^3^ Hebali et al. [33].

**Table 8:** Effect of the different boundary conditions on the non-dimensional central deflection and axial stress of an FGM plate subjected to uniformly distributed load.

		w^5^			σ^5^_xx_(−h/2)			σ^5^_xx_(h/2)		
Condition	*n*	Ref ^1^	Ref ^2^	Present	Ref ^1^	Ref ^2^	Present	Ref ^1^	Ref ^2^	Present
SSSS	0	0.1671	0.1657	0.168	−0.2876	−0.288	−0.31007	0.2968	0.296	0.31007
	0.5	0.2505	0.2482	0.2425	−0.172	−0.1724	−0.171	0.3856	0.384	0.42602
	1	0.2905	0.2878	0.27642	−0.2028	−0.2028	−0.19729	0.424	0.424	0.47441
	2	0.328	0.3251	0.31203	−0.2268	−0.2268	−0.21965	0.468	0.464	0.52509
	Metal	0.4775	0.4734	0.49659	−0.2876	−0.288	−0.29349	0.2968	0.296	0.29349
CCCC	0	0.0731	0.0729	0.073765	−0.1432	−0.1428	−0.16244	0.1592	0.1588	0.16244
	0.5	0.1073	0.1069	0.10755	−0.0844	−0.0848	−0.09724	0.2044	0.2036	0.21235
	1	0.1253	0.1248	0.12507	−0.0992	−0.0992	−0.11496	0.2252	0.2244	0.23515
	2	0.1444	0.1438	0.14404	−0.1116	−0.1112	−0.1287	0.248	0.2464	0.26034
	Metal	0.2088	0.2082	0.07435	−0.1432	−0.1428	−0.13466	0.1592	0.1588	0.13466
SCSC	0	0.1017	0.1009	0.10235	−0.1796	−0.178	−0.22102	0.1848	0.1832	0.22102
	0.5	0.1501	0.1489	0.14892	−0.1052	−0.1044	−0.13065	0.2356	0.2336	0.28952
	1	0.1751	0.1737	0.17238	−0.1244	−0.1232	−0.1536	0.2604	0.2576	0.31981
	2	0.2008	0.1993	0.19756	−0.1408	−0.1396	−0.17148	0.2884	0.2852	0.35333
	Metal	0.2905	0.2882	0.29871	−0.1796	−0.178	−0.19277	0.1848	0.1832	0.19277
SFSF	0	0.5019	0.5015	0.5029	−0.736	−0.728	−0.76655	0.744	0.736	0.76655
	0.5	0.7543	0.7532	0.72096	−0.44	−0.436	−0.41144	0.968	0.96	1.0550
	1	0.8708	0.8693	0.81424	−0.52	−0.516	−0.47007	1.068	1.056	1.1725
	2	0.9744	0.9719	0.91146	−0.584	−0.576	−0.52238	1.176	1.16	1.2974
	Metal	1.4345	1.4330	1.4633	−0.736	−0.728	−0.76655	0.744	0.736	0.76655

^1^ Gilhooley et al. [30] multiquadric, ^2^ Gilhooley et al. [30] thin plate spline.

**Table 9:** Effect of the skewness angles on the non-dimensional central deflection (w6) of the simply supported FGM plate.

			w^6^				
a/h	Skew Angle	Liew and Han [46]	Present (n = 0)	0.5	1	2	5
5	0°	7.8469	7.6173	11.7676	13.4586	15.278	17.707
	15°	7.0945	7.2706	10.5834	12.1196	13.7691	15.9524
	30°	5.1714	5.1624	7.5428	8.6705	9.8819	11.451
	45°	2.8913	2.7207	3.9906	4.6198	5.3082	6.1773
10	0°	6.8365	7.0310	10.2523	11.6278	13.0085	14.8909
	15°	6.1390	6.2924	9.1907	10.4338	11.6753	13.3511
	30°	4.3714	4.3963	6.4473	7.3384	8.2191	9.3786
	45°	2.3191	2.2012	3.2427	3.7063	4.1633	4.7466
100	0°	6.5031	6.4831	9.4922	10.7298	11.9164	13.5406
	15°	5.8236	5.7819	8.4807	9.5956	10.658	12.0959
	30°	4.1054	3.9735	5.8536	6.6391	7.3764	8.3458
	45°	2.1204	1.8936	2.8029	3.189	3.5454	3.9968

**Table 10:** Effect of the skewness angles on the non-dimensional central deflection (w6) of the FGM plate with clamped edges.

		ω^6^				
Skew Angle	Butalia et al. [47]	Present (n = 0)	0.5	1	2	5
15°	1.7948	1.7402	2.3615	2.7551	3.134	3.4992
30°	1.2281	1.185	1.6075	1.8756	2.1347	2.386
45°	0.5997	0.57342	0.77715	0.90698	1.0337	1.1584
60°	0.1704	0.16135	0.21831	0.2549	0.29133	0.32816
75°	0.0143	0.01410	0.01902	0.02224	0.02557	0.02917

## Data Availability

Not applicable.

## Appendix A

$${\left[B\right]}^{T}=\sum_{i=1}^{9}\left[\begin{array}{cccccccccccccccccc} N_{i,x} & 0 & N_{i,y} & 0 & 0 & 0 & 0 & 0 & 0 & 0 & 0 & 0 & 0 & 0 & 0 & 0 & 0 & 0\\ 0 & N_{i,y} & N_{i,x} & 0 & 0 & 0 & 0 & 0 & 0 & 0 & 0 & 0 & 0 & 0 & 0 & 0 & 0 & 0\\ 0 & 0 & 0 & N_{i,x} & N_{i,y} & 0 & 0 & 0 & 0 & 0 & 0 & 0 & 0 & 0 & 0 & 0 & 0 & 0\\ 0 & 0 & 0 & 0 & 0 & N_{i,x} & 0 & N_{i,y} & N_{i} & 0 & 0 & 0 & 0 & 0 & 0 & 0 & 0 & 0\\ 0 & 0 & 0 & 0 & 0 & 0 & N_{i,y} & N_{i,x} & 0 & N_{i} & 0 & 0 & 0 & 0 & 0 & 0 & 0 & 0\\ 0 & 0 & 0 & N_{i} & 0 & 0 & 0 & 0 & 0 & 0 & N_{i,x} & 0 & N_{i,y} & N_{i} & 0 & 0 & 0 & 0\\ 0 & 0 & 0 & 0 & 0 & 0 & 0 & 0 & 0 & 0 & 0 & N_{i,y} & N_{i,x} & 0 & N_{i} & 0 & 0 & 0\\ 0 & 0 & 0 & 0 & 0 & 0 & 0 & 0 & 0 & 0 & 0 & 0 & 0 & 0 & 0 & N_{i} & N_{i,x} & N_{i,y} \end{array}\right]$$

$$\left[H\right]=\left[\begin{array}{cccccccccccccccccc} 1 & 0 & 0 & 0 & 0 & -z & 0 & 0 & 0 & 0 & -f(z) & 0 & 0 & 0 & 0 & 0 & 0 & 0\\ 0 & 1 & 0 & 0 & 0 & 0 & -z & 0 & 0 & 0 & 0 & -f(z) & 0 & 0 & 0 & 0 & 0 & 0\\ 0 & 0 & 0 & 0 & 0 & 0 & 0 & 0 & 0 & 0 & 0 & 0 & 0 & 0 & 0 & g\prime (z) & 0 & 0\\ 0 & 0 & 1 & 0 & 0 & 0 & 0 & -z & 0 & 0 & 0 & 0 & -f(z) & 0 & 0 & 0 & 0 & 0\\ 0 & 0 & 0 & 1 & 0 & 0 & 0 & 0 & -1 & 0 & 0 & 0 & 0 & -f\prime (z) & 0 & 0 & g\prime (z) & 0\\ 0 & 0 & 0 & 0 & 1 & 0 & 0 & 0 & 0 & -1 & 0 & 0 & 0 & 0 & -f\prime (z) & 0 & 0 & g\prime (z) \end{array}\right]$$

$$N\left(\xi ,\eta \right)=\left\{\begin{array}{c} \frac{1}{4}\left(\xi -1\right)\left(\eta -1\right)\xi \eta ,\;\frac{1}{4}\left(\xi +1\right)\left(\eta -1\right)\xi \eta ,\;\frac{1}{4}\left(\xi +1\right)\left(\eta +1\right)\xi \eta ,\\ \frac{1}{4}\left(\xi -1\right)\left(\eta +1\right)\xi \eta ,-\frac{1}{2}\left(1-\xi^{2}\right)\left(1-\eta \right)\eta ,-\frac{1}{2}\left(1+\xi \right)\xi \left(\eta^{2}-1\right),\;\\ -\frac{1}{2}\left(\xi^{2}-1\right)\left(1+\eta \right)\eta ,-\frac{1}{2}\left(\xi -1\right)\xi \left(\eta^{2}-1\right),\left(1-\xi^{2}\right)\left(1-\eta^{2}\right)\; \end{array}\right\}$$
